# The Global Registry for Hereditary Angioedema due to C1-Inhibitor Deficiency

**DOI:** 10.1007/s12016-021-08855-4

**Published:** 2021-03-31

**Authors:** Andrea Zanichelli, Henriette Farkas, Laurance Bouillet, Noemi Bara, Anastasios E. Germenis, Fotis Psarros, Lilian Varga, Noemi Andrási, Isabelle Boccon-Gibod, Marco Castiglioni Roffia, Michal Rutkowski, Mauro Cancian

**Affiliations:** 1 Department of Internal Medicine, ASST Fatebenefratelli Sacco, Luigi Sacco Hospital-University of Milan, Milan, Italy; 2 Hungarian Angioedema Center of Reference and Excellence, Department of Internal Medicine and Haematology, Semmelweis University, Budapest, Hungary; 3 French National Center of Reference for Angioedema, Grenoble Alpes University Hospital, Grenoble, France; 4 Hereditary Angioedema Expertise Centre, Sangeorgiu de Mures, Romania; 5 Mediquest Clinical Research Centre, Sangeorgiu de Mures, Romania; 6 Department of Immunology & Histocompatibility, School of Medicine, University of Thessaly, Larissa, Greece; 7 Naval Hospital, Athens, Greece; 8 Patient Representative Member of the Associazione Volontaria Per L’angioedema Ereditario Ed Altre Forme Rare Di Angioedema A.A.E.E, Naples, Italy; 9 Patient representative member of the Hereditary Angioedema International HAEi, Warsaw, Poland; 10 Department of Systems Medicine, University Hospital of Padua, Padua, Italy

**Keywords:** Hereditary angioedema, Global, Registry, Database, Attacks, C1-inhibitor, Prophylaxis

## Abstract

Hereditary angioedema (HAE) is a rare condition, mostly due to genetic deficiency of complement C1 inhibitor (C1-INH). The rarity of HAE impedes extensive data collection and assessment of the impact of certain factors known to affect the course of this disabling and life-threatening disease. Establishing a global registry could assist to overcome such issues and provides valuable patient data from different countries. The HAE Global Registry is a disease-specific registry, with web-based electronic support, where data are provided by physicians and patients through a dedicated application. We collected data between January 1, 2018, and August 31, 2020. Data on 1297 patients from 29 centers in 5 European countries were collected. At least one attack was recorded for 497 patients during the study period. Overall, 1182 patients were diagnosed with HAE type 1 and 115 with type 2. At the time of database lock, 389 patients were taking long-term prophylactic medication, 217 of which were on danazol. Most recorded attacks affected the abdomen, were generally moderate in severity, and occurred in patients who were not on prophylactic treatment (70.6%, 6244/8848). The median duration of attacks was 780 min (IQR 290–1740) in patients on prophylactic medication and 780 min (IQR 300–1920) in patients not on continuous prophylactic medication. In conclusion, the establishment of a registry for C1-INH-HAE allowed collection of a large amount of data that may help to better understand the clinical characteristics of this disease. This information may enhance patient care and guide future therapeutic decisions.

## Introduction

Angioedema is a localized, self-limiting swelling of the extremities, face, genitals, gastrointestinal tract, and upper airway mucosa. Although it is a common symptom of allergic reactions or chronic urticaria (generally with wheals), patients rarely have angioedema in the absence of wheals. In these cases, angioedema can be acquired or hereditary. A consensus meeting held in 2012 gave the first classification of angioedema without wheals [1]. Hereditary angioedema due to C1-inhibitor deficiency (C1-INH-HAE) is the best-characterized and most common form of HAE. Although this rare disease is still underdiagnosed, the estimated prevalence reported in the literature is 1:50,000 [2]. C1-INH-HAE has a well-defined pathogenetic mechanism and several approved therapies [3].

Despite these achievements, the lack of aggregated data makes the epidemiologic aspects vague. We do not know whether C1-INH-HAE patients have specific comorbidities compared with those in the general population. C1-INH-HAE is characterized by different clinical phenotypes [4], but we cannot stratify patients based on their phenotype because the frequency, duration, and severity of angioedema symptoms have not been prospectively collected in a large population under strict, predefined criteria. Disease registries were created to address these needs.

The use of registries started in the 1930s in the UK and USA as a means to systematically collect data on cancer patients [5]. A patient registry is defined as an organized system that collects, analyzes, and disseminates the data and information on a group of people defined by a particular disease, condition, exposure, or health-related service, and that serves a predetermined scientific, clinical, or/and public health (policy) purpose [6]. Depending on the main inclusion criteria, registries are categorized as disease or condition registries, product registries, and health services registries. Product registries for C1-INH-HAE were developed as part of post-marketing surveillance for newly approved treatments [7, 8]. Some of these registries also aim to capture epidemiological data on disease course, but with the recruitment bias of being treatment-oriented [9, 10]. Country-based databases and registries also exist but have limitations in the number of entries and recruited patients [11–13]. An attempt to create a European registry started in 2002 but could not be maintained [14]. Here, we present the design and initial data from the first Hereditary Angioedema Global Registry (HGR).

The objective of this international multicenter disease registry is to gather homogeneous laboratory and clinical data on patients with hereditary angioedema and to evaluate therapeutic options to manage this disease. The competent board (Comitato Etico Indipendente Milano Area A, Registro Sperimentazioni n. 2015/ST/253) of the coordinating center in Milan approved the registry protocol as an investigator-initiated observational study in 2016 and the amended version on February 27, 2017.

## Materials and Methods

The principal investigator at each center was responsible for obtaining a competent ethics committee/institutional review board approval. Each of them identified other co-investigators who collect patients’ informed consent and insert their data in the registry. Upon acceptance of the informed consent, the treating physician transferred data of the case report form (CRF) to a specifically designed electronic form (eCRF). Physicians filled the eCRF with data about the center and record the entry in the registry. Data included demographics; contact details; date and age of diagnosis; type of HAE; levels of functional and antigenic C1-INH, C4, C1q, and anti-C1-INH antibodies; and family history of HAE. Data on *SERPING1* gene transcript, protein mutations, comorbidities, long-term prophylaxis, follow-up, and attacks was also entered. The *SERPING1* genotype was labeled according to the last HUGO nomenclature [15]. Conversion from other nomenclatures was obtained by direct connection with the Mutalyzer software [16]. In addition to signing a specific informed consent, patients consented to long-term storage of biological material (plasma and DNA) for research purposes. Only protocol-related data were collected. To normalize differences in reference values, diagnostic parameters were transformed into a percentage of normal by a converter.

**Fig. 1:**
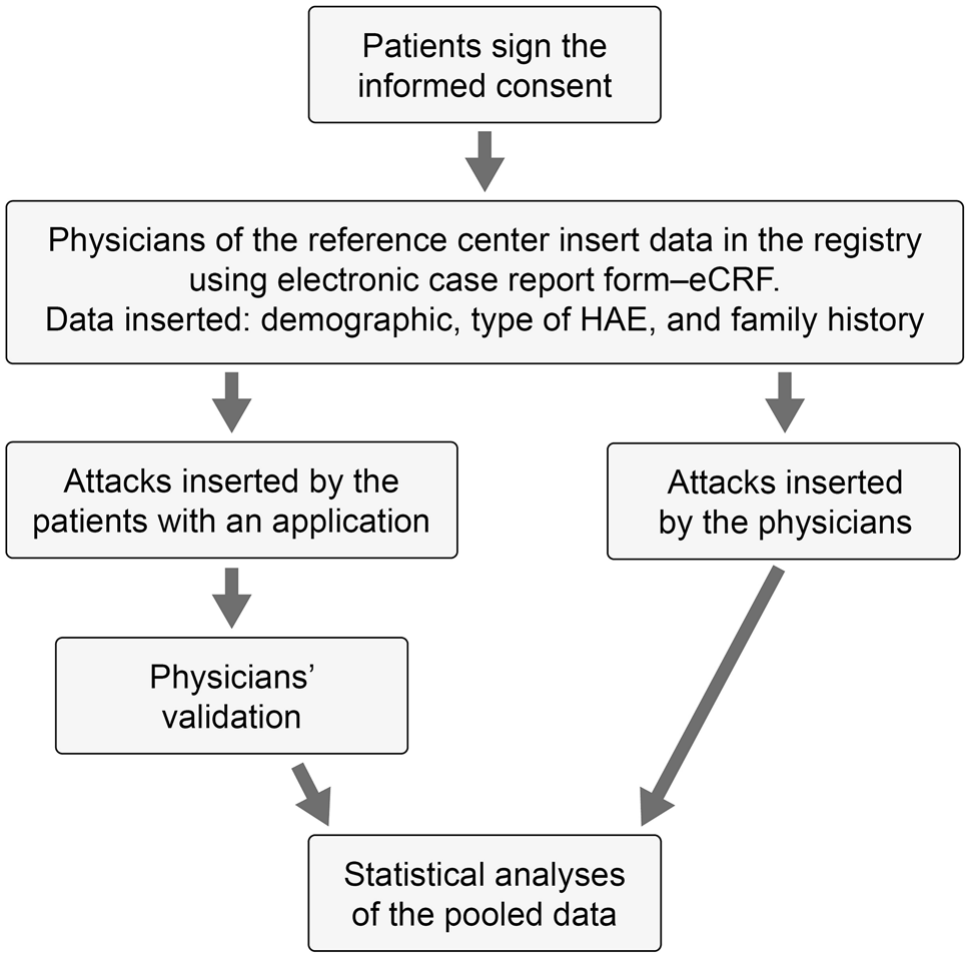
Flowchart explaining how the registry works

When eCRF for a specific patient has been built, the patient him/herself could directly provide data on angioedema (AE) attacks (i.e., duration, severity, and treatment of each angioedema episode) either on paper, using a web form, or by a mobile application (App). These data were directed to a staging area for physician validation before being considered amenable for statistics (Fig. 1).

While the registry contained some retrospective historical values, such as laboratory data, age at diagnosis, all real-time data on the attacks was collected prospectively. Therefore, this study can be considered primarily a prospective cohort study.

The registry governance was approved during the first HGR Conference held in Bucharest, on April 13, 2018. An independent non-profit foundation (HGRF), made of representatives of patients’ associations, was in charge of funding and delegated all the management to the HAE Global Registry Board (HGRB). The HGRB oversees operational tasks, assisted by the HAE Global Registry Scientific Committee (HGRSC) which supervises issues of competence. No registry member, center, group, or board could access the entire set of data. All registry members, as single or group, could propose new studies based on the aggregated data, by addressing their request to the HGRSC. Analysis and studies based on data obtained at local centers was allowed at any time. Members of the HGRB and HGRSC were elected to be representatives of different cultural and geographic backgrounds. Their offices have a 2-year term with no more than one consecutive renewal. Angioedema centers could join the registry upon request from the HGRB. The registry quality-control system periodically checks registry entries and compliance of eCRF with the source data. For each dataset, the system could trace the time and author.

We collected data on living C1-INH-HAE type 1 or type 2 patients from all participating centers. The diagnosis of C1-INH-HAE type 1 and 2 was based on the following criteria: C1-INH-HAE type I was diagnosed when functional and antigenic C1-INH were ≤ 50% of normal, and type II when functional C1-INH was ≤ 50% and antigenic C1-INH was > 50% of normal. Normal values were 70–130% for functional C1-INH, 70–115% for antigenic C1-INH, and 60–140% for antigenic C4.

The following patients’ data were included in this study: number of patients per center, age, gender, HAE diagnosis, number of patients recording at least one attack, the tool used for recording the attacks, and the number of recorded patients during the study period. In addition, the distribution of patients taking long-term prophylactic medication according to the type of prophylaxis, gender, and age was extracted, using the ongoing situation at the time of database lock, that is, on August 31, 2020.

Data on valid attacks occurring from January 1, 2018, to August 31, 2020 were included in this analysis. We recorded the total valid attacks, attack location, severity, duration, and the use of prophylactic treatment. We assessed the severity of attacks, according to their interference with activities of daily living, as: ‘*mild*’ if no interferences were experienced, ‘*moderate*’ in case of partial interference, and ‘*severe*’ for complete incapacity.

## Results

### Patients

**Fig. 2:**
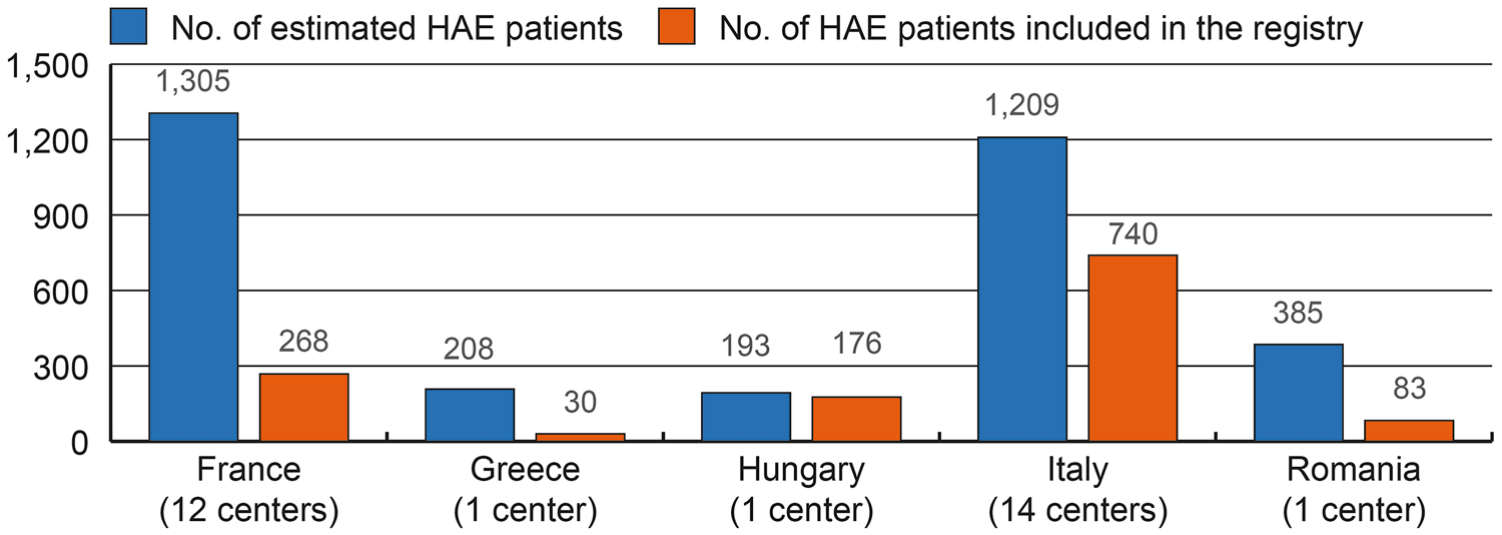
Total number of estimated HAE patients and number of HAE patients recorded in the registry. The estimations of the total affected population were based on 1:50,000 rate [2] to the most updated population data in each country [17]

At the end of the analyzed 32-month period, the total number of living patients in the registry was 1297, from 29 centers, most of which (> 50%) were from Italy (Fig. 2). It is interesting to note that the Hungarian center encompassed almost the entire Hungarian estimated patient population.

The registry’s patient disposition is presented in Table 1. Approximately 12% of the recorded patients were under age 18. The number of females is slightly higher than males (56%). The most represented type of HAE is type 1, accounting for 91% of patients. The median age at diagnosis was 23 years, but some were not diagnosed until the age of 80.

Of the entire patient population in the registry, 829 (64%) were holding an account enabling them to register attack characteristics by themselves. Among those who had the account and recorded at least one attack (*n* = 410), 86% registered the attack by themselves, while 14% of attacks were recorded by physicians.

From the beginning to the end of the analysis period, the number of recorded patients has almost tripled. Overall, less than 50% of patients in the database had at least one recorded attack. The growth rate of the database is depicted in Table 1. Laboratory data at diagnosis, when available, is reported in Table 2.

**Fig. 3:**
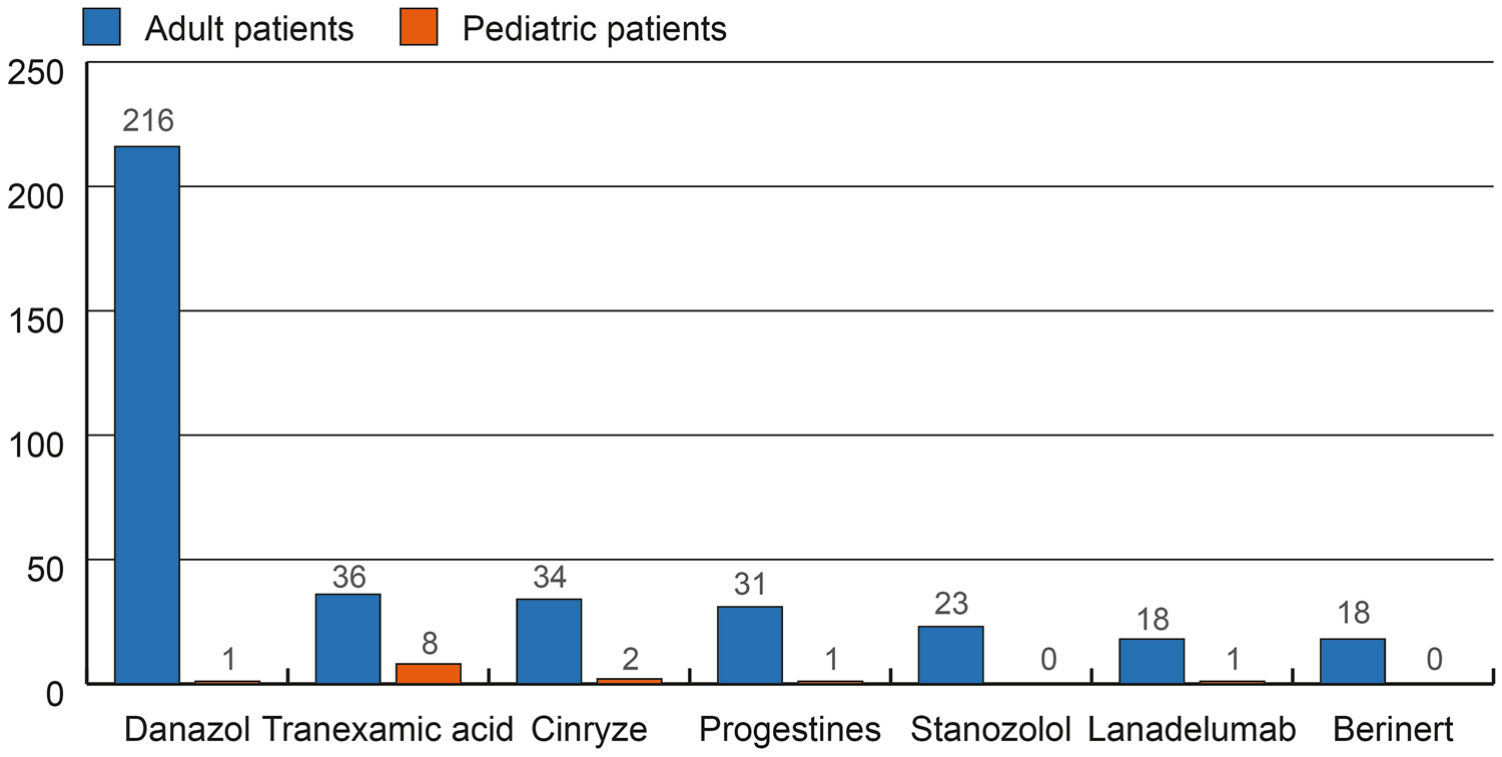
Adult and pediatric patients taking long-term prophylaxis (LTP) at the time of database lock. Pediatric patients were < 18 years of age. Adult patients were ≥ 18 years of age, Berinert: I.V. and S.C. are considered off-label

Until the data-cut on the 31st of August 2020, 389 patients were taking long-term prophylactic medication, of the majority were using attenuated androgens like danazol (Fig. 3), which was also administered in a high percentage of female patients (45%, Table 3). Interestingly, 40 out of 240 (17%) patients taking androgens (danazol or stanozolol) at the database lock have started using it during the study period.

### Recorded Angioedema Attacks

In the period considered, the total validated attacks were 8848, attributable to 497 patients (Table 4). Most attacks (71%) occurred in patients not on prophylactic medication. The most frequently affected sites were the abdominal area and the skin. The severity of attacks was considered moderate, and attack duration was similar in patients with and without prophylaxis (Table 4).

## Discussion

A global registry for HAE permits collection of valuable data from many patients affected by this rare disease across countries and cultures. The implementation of this registry was possible thanks to the first initiative of the Italian Network for C1-INH-HAE (ITACA) established in 2012, which in 2018 decided to expand its data collection framework to other European countries: Greece, Hungary, France, Romania [18]. The first attempt to form an European HAE registry was in 2002, involving centers from Italy, Germany, Hungary, Denmark, France, Spain, the UK, Norway, Poland, and Switzerland [14]. However, this enterprise was not maintained for many reasons. Other similar registries, involving patients with HAE, were also attempted. For example, a product registry for patients using Berinert (pdC1-INH, CSL-Behring) was created and reported in 2016 [8]. In addition, country-based registries also collected patient data. In Greece, Psarros et al. were able to gather data and report on 116 patients [11]. However, data collected by the Greek registry was in an eCRF form but were not monitoring the progression of attacks and changes in drug treatment. The Spanish registry, reported by Roche and colleagues [13], also recorded long-term prophylaxis (LTP), highlighting a high prevalence of attenuated androgen use. However, it is worth noting that this report was published in 2005 when drug options were limited. In the meantime, global experience (i.e., Canada, Brazil, International guidelines) has been published [19, 20].

### Type of HAE

The proportion of patients affected by C1-INH-HAE type 1 and type 2 enrolled in this study was 91% and 9%, respectively, which is in-line with data reported by others in the literature [2, 21]. In comparison, nationwide surveys carried out in Italy [2] and Denmark [21] found prevalence of C1-INH-HAE type 1 of 87% and 94%, respectively.

### Attack Location

The most common site of attack in this study was the abdomen. A survey carried out in Spain, Germany, and Denmark [22] also reported that the abdominal area (or intestinal involvement) was the most common site of attack.

### Long-Term Prophylaxis

In this study, 30% of patients were maintained on LTP at the database lock. Bygum and colleagues, in a Danish nationwide survey, found a similar proportion (29%) [21]. It is worth noting that the most commonly used drug for LTP was attenuated androgens. These drugs have some advantages, such as low price and easy (oral) administration, but this is at a cost of many adverse effects [23]. In recent years, new targeted drugs carrying a lower risk of adverse effects have been approved as LTP for C1-INH-HAE. In this database, most patients treated with danazol and stanozolol began their prophylaxis therapy many years ago, and only 17% started using it during the study period.

### Duration of Attacks

The duration of attacks with or without prophylaxis was similar. This might be due to the fact that patients with severe disease preferred to use prophylaxis because it shortens the duration of attacks, or due to the fact that patients without prophylaxis promptly treated acute attacks with on-demand therapy.

### Real-Time Recording

Among the other advantages of this specific registry is an easy-to-use application (App) in the smartphones of the patients. This enables a prompt recording of the characteristics of the attack and alerts physicians about self-treatment. This way, the patients may timely observe the efficacy of on-demand treatment, disease severity, and the need for starting prophylaxis or switching to another drug. Among the other advantages, the registry could capture data about a switch in prophylactic treatment and monitor the use of new drugs. It should be noted that the recent market entry of the anti-kallikrein monoclonal antibody lanadelumab [24, 25], the data about its use is represented only in the last year of the study period.

### Limitations

This registry also has some limitations. First, the completeness of the data on recorded attacks is a matter of concern, as only 497 patients reported ≥ 1 attack. Of these patients, 359 (72%) recorded the attack by the App. The attacks from the remaining 138 were inserted by physicians. No attacks were recorded for 800 patients, thus raising some doubts regarding patient compliance. Another critical issue is the validation of attacks by physicians, which may result in a workload not always sustainable without adequate staff support.

Second, the registry needs to be fed continuously. So far, few European countries participate in this project, and the involved countries are far from covering the whole national patient population. Several HAE reference centers, even in the participating countries, did not provide data to this registry. Hungary is an exception, as it seems to have included the larger majority (91%) of the evaluated patients.

Finally, the dates of the first onset of symptoms were not captured, and dates about the diagnosis alone do not allow us to know what was the cause of delay in diagnosis. We can only calculate the mean delay of the diagnosis from the reference age of 20 years, as it was shown that 85% of HAE patients were already symptomatic during the first two decades of life [26]. The delay in diagnosis for HAE is estimated to range between 12 and 16.3 years from the first onset of symptoms [10, 13, 21, 27]. According to these presumptions, the mean delay in the diagnosis found in our study was circ. 5 years.

## Conclusions

The establishment of a global registry for C1-INH-HAE allows physicians to collect and analyze a large amount of data about this rare disease. In the near future, a new version of the registry with additional tools is planned. This instrument will enable physicians to perform more analyses and obtain better understanding of the natural course of C1-INH-HAE, as well as evaluate quality-of-life and effectiveness of on-demand and prophylactic treatments.

**Table 1:** Data recorded in the registry in the period of the study

Charachteristics	N (%; median; range; IQR [years of age])
Pediatric^a^ patients	140 (11%; 11; 2-17; 8-14)
Adult^b^ patients	1,157 (89%;46; 18-92; 32-59)
Gender	N (%)
Males	569 (44%)
Females	728 (56%)
Type of C1-INH-HAE	N (%)
Type 1	1,182 (91%)
Type 2	115 (9%)
Age at diagnosis (n=1295)	(mean; median; range; IQR)
Age at diagnosis (years)	(25; 23; 0-80; 10-36)
No. of patients inserted attacks during the study period	
Patients with ≥1 attack	497
Attack inserted by the app	359
Attack inserted by a physician	138
No. of patients recorded during the study period	
Up to January 2018	440
Up to January 2019	790
Up to January 2020	1,129

^a^<18 years of age, ^b^≥18 years of age, *IQR* = interquartile range

**Table 2:** Laboratory data at diagnosis in HAE type 1 (n=1,182) and 2 (n=115)

Parameter	C1-INH-HAE type 1 (mean; median; IQR)	C1-INH-HAE type 2 (mean; median; IQR)
C4 (% of normal^a^)	18; 15; 1–26	19; 18; 3–26
Antigenic C1-INH (% of normal^b^)	20; 20; 9–26	107; 108; 53–156
C1-INH function (% of normal^c^)	20; 17; 2–30	20; 18; 9–30

^a^60 to 140%, ^b^70 to 115%, ^c^70 to 130%

**Table 3:** Drugs taken for long-term prophylaxis (LTP)

Drugs	Total	Pediatric patients^a^		Adult patients^b^	
		M	F	M	F
Danazol	217	0	1	118	98
Tranexamic acid	44	6	2	11	25
Cinryze	36	1	1	8	26
Progestines	32	0	1	0	31
Stanozolol	23	0	0	4	19
Lanadelumab	19	1	0	9	9
Berinert^c^	18	0	0	2	16
Total	389	8	5	152	224

^a^<18 years of age, ^b^≥18 years of age, ^c^I.V. and S.C. Off-label use

**Table 4:** Location, severity, and duration of attacks in the period considered.

	Attacks with prophylaxis (*n*=2641)	Attacks without prophylaxis (*n*=6244)
Site^a^		
Cutaneous neck/face	185 (6%)	404 (6%)
Abdominal	1277 (40%)	3017 (42%)
Cutaneous others	1279 (40%)	2759 (38%)
Oral cavity/larynx	82 (3%)	202 (3%)
Other sites (including genital attacks)	368 (12%)	867 (12%)
Severity		
Mild	759 (29%)	1568 (25%)
Moderate	1333 (50%)	3206 (51%)
Severe	549 (21%)	1470 (24%)
Duration		
Time in minutes (median; IQR [min])^b^	780; 290–1740	780; 300–1920

Some attacks affected more than one site, valid attacks in the period (01 Jan 2018<= Start Date<=31 Aug 2020) attack duration < 12 days (22 records were excluded because attack duration was >12 days). Percentages represent the proportions within the same category (i.e., with prophylaxis/without prophylaxis)

## Data Availability

Data available upon request from the authors.

## Abbreviations

C1-INH: C1 inhibitor
C1-INH-HAE: Hereditary angioedema due to C1-Inhibitor deficiency
CRF: Case-report form
eCRF: Electronic case report form
HAE: Hereditary angioedema
HGR: HAE Global Registry
HGRB: HAE Global Registry Board
HGRSC: HAE Global Registry Scientific Committee
IQR: Interquartile range
LTP: Long-term prophylaxis

## Declarations

### Disclaimer

The funders had no role in the study design, data collection, and analysis, decision to publish, or preparation of the manuscript.
